# Rectal complications following SpaceOAR insertion after prior pelvic radiation

**DOI:** 10.1093/bjrcr/uaaf013

**Published:** 2025-03-17

**Authors:** Andrew H Yates, Philip J Dempsey, Jack W Power, Adam Agnew, Brian D Murphy, Calvin Coffey, Richard Moore, Mazen El Bassiouni, Michelle M J McNicholas

**Affiliations:** Radiology Department, The Mater Hospital, Dublin D07 R2WY, Ireland; Radiology Department, The Mater Hospital, Dublin D07 R2WY, Ireland; Radiology Department, The Mater Hospital, Dublin D07 R2WY, Ireland; Radiology Department, The Mater Private Hospital, Dublin D07 WKW8, Ireland; Radiology Department, The Mater Private Hospital, Dublin D07 WKW8, Ireland; School of Medicine, University of Limerick, Limerick V94 T9PX, Ireland; Radiology Department, The Mater Private Hospital, Dublin D07 WKW8, Ireland; Mater Private Network, Mid-Western Radiation Oncology Centre, Dooradoyle, Limerick V94 F858, Ireland; Radiology Department, The Mater Hospital, Dublin D07 R2WY, Ireland

**Keywords:** prostate cancer, hydrogel spacer, SpaceOAR, pelvic radiation, radiation therapy, complications, retrospective case series

## Abstract

Treating prostate cancer with radiation therapy in patients with a history of prior pelvic radiation may be limited by rectal dose constraints and the risk of rectal toxicity. Rectal spacers have been shown to improve rectal dosimetry in the treatment of prostate cancer. This study aimed to evaluate the safety and outcomes of hydrogel spacer placement, specifically SpaceOAR, between the rectum and prostate in prostate cancer patients who had previously undergone radiation therapy. In this retrospective case series, we analysed the medical records of 8 sequential patients undergoing reirradiation in the setting or prior pelvic radiation, who had received transperineal SpaceOAR placement. We documented the incidence of complications after SpaceOAR placement, before and after undergoing radiation therapy. There was a spectrum of complications in this patient cohort, ranging from pelvic pain to more severe complications such as rectal perforation abscess and fistula. Severe complications occurred in 2 of the 8 patients. Re-irradiation may increase the risk of normal tissue complications; however, hydrogel spacer placement using SpaceOAR in prostate cancer patients with prior pelvic radiation was associated with a higher rate of rectal complications than expected in a small series of patients. We urge caution when using SpaceOAR in this patient group.

## Introduction

Rectal spacers have an excellent safety profile in patients without prior pelvic radiation, in external beam and brachytherapy, and are effective in improving rectal radiation dosimetry by increasing the distance between the rectum and the prostate and significantly decreasing the rate of grade 2 or higher rectal toxicity.^1^^,^^2^ The authors have had extensive experience with the product SpaceOAR (Boston Scientific Corporation, Marlborough, MA, USA) in a high-volume centre of excellence with nearly 700 cases performed to date and have published on its excellent safety profile in the radiation naïve population.^3^^,^^4^

Radiation of the prostate in the setting of prior pelvic radiation is limited by rectal dose constraints. Prostate reirradiation using brachytherapy has been shown to have favourable safety profiles.^5^^,^^6^ Increasing the distance between the rectum and the prostate can be achieved with an implanted spacer made from substances including polyethylene-glycol hydrogel and hyaluronic acid.^7^ Increasing the distance between the rectum and prostate can also be achieved by inserting a biodegradable balloon spacer.^8^ In theory, a rectal spacer should benefit this patient cohort in particular by increasing the space between the prostate and the rectum, reducing the risk of late rectal complications.

Prior radiation causes fibrosis and altered vascularisation of all affected tissues.^9^ This may affect the safety and delivery of the spacer. Potential late effects of radiation on pelvic anatomy and tissue integrity could elevate the risk of complications such as infection or spacer misplacement and may also increase procedural difficulty. We reviewed a series of cases detailing our experience with SpaceOAR placement in men who had previously undergone pelvic irradiation. Informed consent was obtained from the patients for publication of this case series, including accompanying images.

## Case series

Our unit has extensive experience in delivering SpaceOAR to men undergoing radiotherapy for de novo prostate cancer and has demonstrated this technique to be safe and well-tolerated.^3^ From April 2021, SpaceOAR was placed in 8 patients with prostate cancer who had prior pelvic radiation therapy over a 5-month period. All patients had standard prostate cancer work-up, including tumour marker (PSA), MRI, and CT imaging. Biochemical failure was diagnosed using the Phoenix Criteria.^10^ Patients were clinically assessed and discussed at a multidisciplinary meeting before referral for the procedure. Patients were deemed technically suitable for SpaceOAR if there was no gross extracapsular extension of the tumour into the retro-prostatic space. Seminal vesical involvement was not considered a contraindication. Written informed consent was obtained from the patients for publication of this case series, including accompanying images.

This review examines the clinical outcomes of 8 consecutive patients who underwent transperineal hydrogel spacer placement before radiotherapy for prostate cancer. Patients’ medical records were reviewed to assess adverse events after hydrogel insertion and after radiation therapy. Adverse events were graded using Common Terminology Criteria for Adverse Events (CTCAE; Version 5.0).^11^

Seven patients had been treated previously for prostate cancer. One patient had prior radiation therapy and surgery for rectal cancer. All patients had confirmation of prostate cancer, or recurrence, by transperineal or transrectal prostate biopsy. All 8 patients had SpaceOAR hydrogel insertion before radiation therapy for prostate cancer.

Three patients had no symptoms after hydrogel placement and no complications during their course of treatment (Table 1). Five of 8 patients had complications ranging from mild to severe (Table 1). One patient developed a severe complication with the formation of a recto-urethral fistula requiring a de-functioning colostomy. One patient developed a small rectal abscess after SpaceOAR which initially responded to antibiotics but subsequently developed a recurrent abscess and osteomyelitis of the pubic bone 3 months after completion of radiotherapy. One patient developed rectal bleeding and proctitis after SpaceOAR and radiotherapy, which eventually settled. One patient developed nocturia and urinary incontinence after completion of radiation treatment. He was found to have a urethral stricture. Symptoms improved after dilation of the stricture. A further patient had urinary symptoms after hydrogel spacer injection and radiation therapy not requiring intervention.

**Table 1.** Patient demographic, radiation history, and complications.

Patient	Initial cancer	Radiation history (external beam radiation—EBRT)	Prostate cancer staging at re-irradiation	Time between radiation treatment	Re-irradiation dose	Volume re-irradiated (V 100%)	Cumulative rectal dose (D2cc) (α/β = 3.0 for OAR)	Complications after SpaceOAR injection before radiation therapy	Complications after radiation therapy CTCAE grade	Current status
1	Prostate adenocarcinoma, T3bN0M0, PSA 42, Gleason 7. 52 cc gland	78 Gy/39 fractions IMRT	16/17 cores +, Gleason 3 + 4 = 7, involving SV’s	12 years	27 Gy/2 fractions	63.6 cc	121.6 Gy	Perineal pain in the days following the SpaceOAR injection treated with oral antibiotics	Recto-urethra fistula requiring a defunctioning colostomy. Grade 4	Died from progressive lung and bone metastases 1 year after radiation therapy
2	T3aN1bM0 rectal cancer	50.4 Gy/28 fractions 3D EBRT followed by anterior resection and low rectal anastomosis	T1cN0M0, PSA 4.3, 11/12 cores +, grade group 2, 42 cc gland	7 years	60 Gy/20 fractions	98.4 cc	115.6 Gy	Pelvic pain and urinary urgency Small rectal intramural injection of the SpaceOAR on MRI	Defect in the anterior rectal wall communicating with a collection between the rectum and the prostate managed with a diverting loop ileostomy and anterior rectal wall repair Pubic symphysis osteomyelitis treated with intravenous antibiotics Grade 4	Currently well 24 months after having recently undergone reversal of ileostomy Pelvic abscess resolved
3	Prostate adenocarcinoma, T2cN0M0, PSA 2.8, Grade group 3, Gland size unknown	60 Gy/20 fractions 3D EBRT	8/15 cores +, psa 2.4, Grade group 4	4 years	27 Gy/2 fractions	30.8 cc	82.8 Gy	No immediate complications	Mild proctitis Urinary symptoms Grade 3	Asymptomatic No recurrence 24 months after radiation therapy
4	Prostate adenocarcinoma, T2N0M0, PSA 6.6, Grade group 2, right gland, gland size unknown	70 Gy/35 fractions 3D EBRT	Right and left prostate and SV positive for grade group 4 disease	18 years	27 Gy/2 fractions	60.8 cc	91.9 Gy	No immediate complications	Urethra stricture requiring dilatation Grade 3	Asymptomatic No recurrence 24 months after radiation therapy
5	Prostate adenocarcinoma, T1cN0M0, PSA < 10, Grade group 2, gland size unknown	LDR implant, 145 Gy (Iodine^125^)	3/6cores from right SV	6 years	27 Gy/2 fractions	10.2 cc	125.8 Gy^12^	No immediate complications	Mild urinary symptoms Grade 1	Asymptomatic No recurrence 24 months after radiation therapy
6	Prostate adenocarcinoma, T2N0M0, PSA 9.8, Grade group 3Gland size unknown	81 Gy/45 fractions IMRT	Left gland, Grade Group 4	10 years	27 Gy/2 fractions	11.7 cc	86.9 Gy	Mild pelvic discomfort	Mild pelvic discomfort Grade 1	Asymptomatic No recurrence 24 months after radiation therapy
7	Prostate adenocarcinoma, T1cNxMx, PSA 3.49, Grade group 1, Gland size unknown	74 Gy/37 fractions 3D EBRT	Biopsy results unavailable, PSMA confirmed local prostatic reoccurrence	9 years	27 Gy/2 fractions	91.7 cc	111.3 Gy	No immediate complications	No complications	Asymptomatic No recurrence 24 months after radiation therapy
8	Prostate adenocarcinoma, T2N0M0, PSA 15, Grade Group 3, 63 cc gland	78 Gy/39 fractions IMRT	5/14 cores, left gland only	12 years	27 Gy/2 fractions	11.0 cc	87.5 Gy	No immediate complications	No complications	Asymptomatic No recurrence 24 months after radiation therapy

### Patient 1

This 72-year-old male had bilateral high-volume prostate cancer recurrence 12 years after initial treatment with external beam radiation therapy (EBRT). On biopsy, 16/17 cores were positive with seminal vesical involvement. There was no evidence of extracapsular spread posterior to the prostate gland. He was considered suitable for SpaceOAR. Hydro-dissection was first performed in the usual manner, but the space created by hydro-dissection was limited compared with that seen in radiation-naive patients. The patient was not symptomatic apart from pressure symptoms after the gel placement. Following the procedure, there was no evidence of complication on MRI and the patient was treated with high-dose brachytherapy 2 weeks later, in 2 fractions 1 week apart.

**Figure 1.**
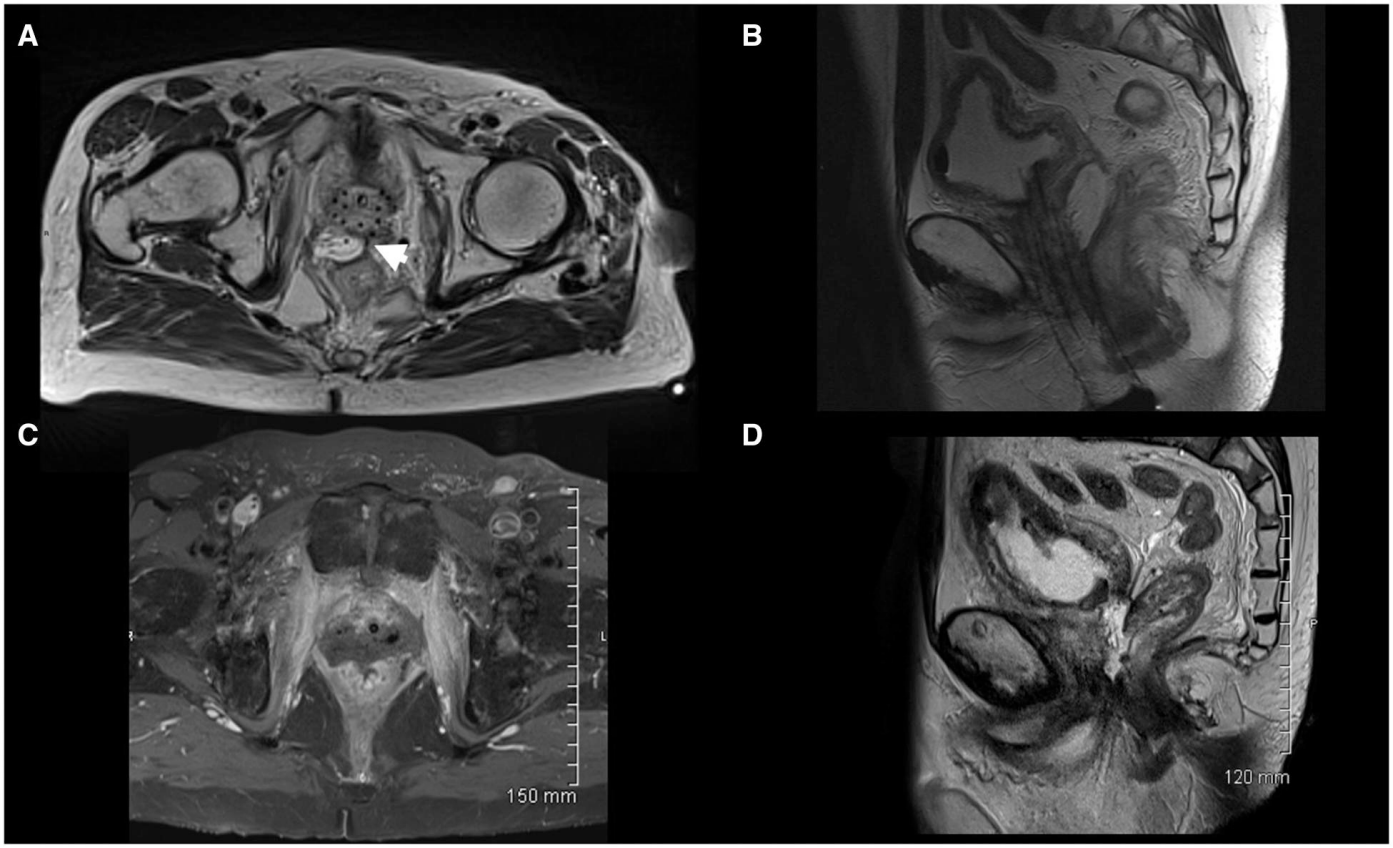
(A and B) Selected axial and sagittal images from a T2 weighted MRI pelvis completed after insertion of the brachytherapy wires showing the hydrogel spacer to be positioned between the rectum and the prostate. The spacer has an asymmetrical distribution with more of the hydrogel on the right. A small adhesion is seen between the prostate and the rectum (arrow head). (C and D) Selected axial T1 weighted images with intravenous contrast and T2 weighted images from an MRI pelvis completed 1 month after brachytherapy was completed. These images show a recto-urethral fistula. There is a defect in the anterior rectal wall communicating with a collection between the rectum and the prostate. The collection is continuous with the prostatic urethra (urinary catheter in situ).

An MRI of the pelvis completed after insertion of the brachytherapy catheters showed the spacer to be positioned between the rectum and the prostate. The spacer had an asymmetrical distribution with more of the hydrogel on the right. A small adhesion was seen between the prostate and the rectum (Figure 1A).

A month after brachytherapy was complete, the patient was admitted complaining of passing urine through the rectum. An MRI of the pelvis revealed a defect in the rectal wall communicating with the SpaceOAR cavity, which contained gas, and a communication between the SpaceOAR cavity and the prostatic urethra (Figure 1C and D). The patient was examined endoscopically. This confirmed the presence of a recto-urethral fistula. The patient subsequently underwent a de-functioning colostomy and urethral repair and had a long-term suprapubic urinary catheter inserted. The complications were categorized as adverse events (life-threatening consequence requiring urgent intervention).

Eight months after completion of treatment, his condition worsened. Follow-up imaging found local progression of prostate cancer with the development of lung and bone metastases. The metastatic lung disease was rapidly progressive, and the patient died 3 months later.

### Patient 2

**Figure 2.**
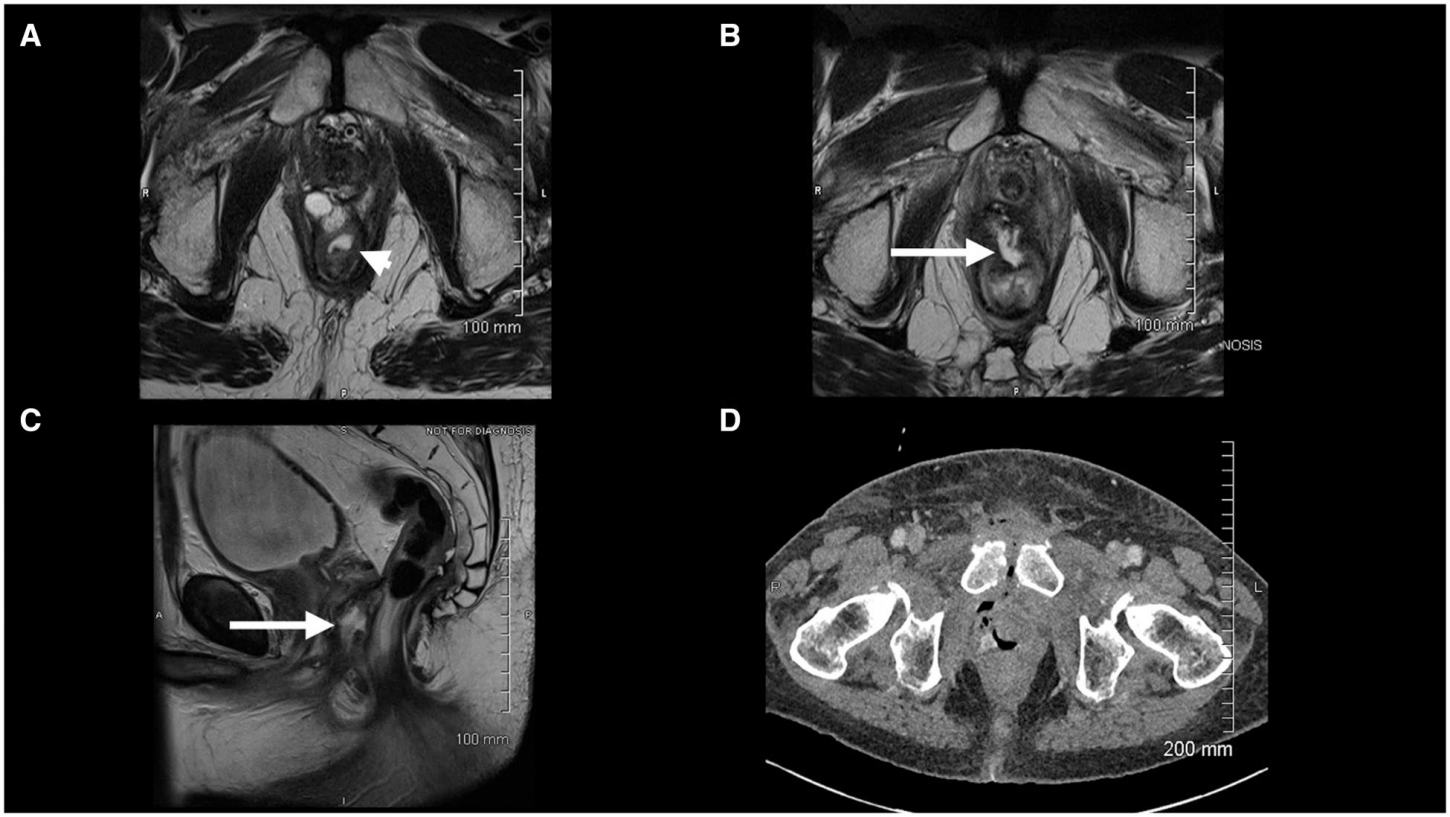
(A) Selected axial T2 weighted images from an MRI pelvis after hydrogel injection and before radiation therapy showing a collection within the anterior rectal wall (arrow head). (B and C) Selected axial and sagittal images from an MRI pelvis after radiotherapy treatment showing an enlarging defect in the anterior rectal wall communicating with a collection between the rectum and the prostate in keeping with an abscess (arrow). (D) Selected axial image from a CT abdomen and pelvis with intravenous contrast showing the collection containing fluid and air to track anteriorly in the pelvis and involve the pubic symphysis with cortical erosion consistent with osteomyelitis.

A 79-year-old male had a diagnosis of bilateral prostate cancer 7 years after prior chemoradiotherapy and surgery for rectal cancer. He had an anastomotic stricture (2 cm lumen) that allowed passage of the transrectal probe and had undergone ultrasound-guided prostate biopsies for diagnosis of prostate cancer without difficulty. As he had tolerated transrectal biopsy, he was considered suitable for SpaceOAR. Hydrodissection was possible but created only a small space. Insertion of the gel was terminated after 5 mL was injected because of high pressure. The patient had local pressure symptoms and pain immediately after injection of the SpaceOAR. A pelvic MR was performed that documented a small volume (approximately 1 mL) of the spacer in the anterior rectal wall (Figure 2A). The patient was managed conservatively with antibiotics and his symptoms resolved over the course of 4 weeks.

The patient underwent EBRT to the prostate 1 month after insertion of the SpaceOAR, 60 Gy delivered over 20 sessions. Three months following the completion of radiation therapy, he presented with perineal pain and swelling. MRI demonstrated a defect in the low rectal wall with abscess formation in the pelvis (Figure 2B and C). The patient was managed conservatively as an outpatient with antibiotics, but although symptoms improved initially, he presented again with rectal discharge and pelvic pain six months later. Imaging studies, including an MRI and CT, showed a persistent defect in the rectal wall. A collection tracking anterior to the prostate in the pelvis and osteomyelitis of the pubic symphysis (Figure 2D). He underwent an anterior rectal wall repair and loop ileostomy and was treated with intravenous antibiotics. The ileostomy has subsequently been reversed and follow-up imaging demonstrated that the pelvic collection has resolved. The complications were categorized as a Grade 4 adverse event (life-threatening consequence requiring urgent intervention).

### Patient 3

A 79-year-old male with organ-confined Gleason 7 prostate cancer recurrence 4 years after his initial treatment with EBRT. He underwent injection of a hydrogel SpaceOAR without issue before undergoing salvage brachytherapy of 27 Gy over 2 sessions.

**Figure 3.**
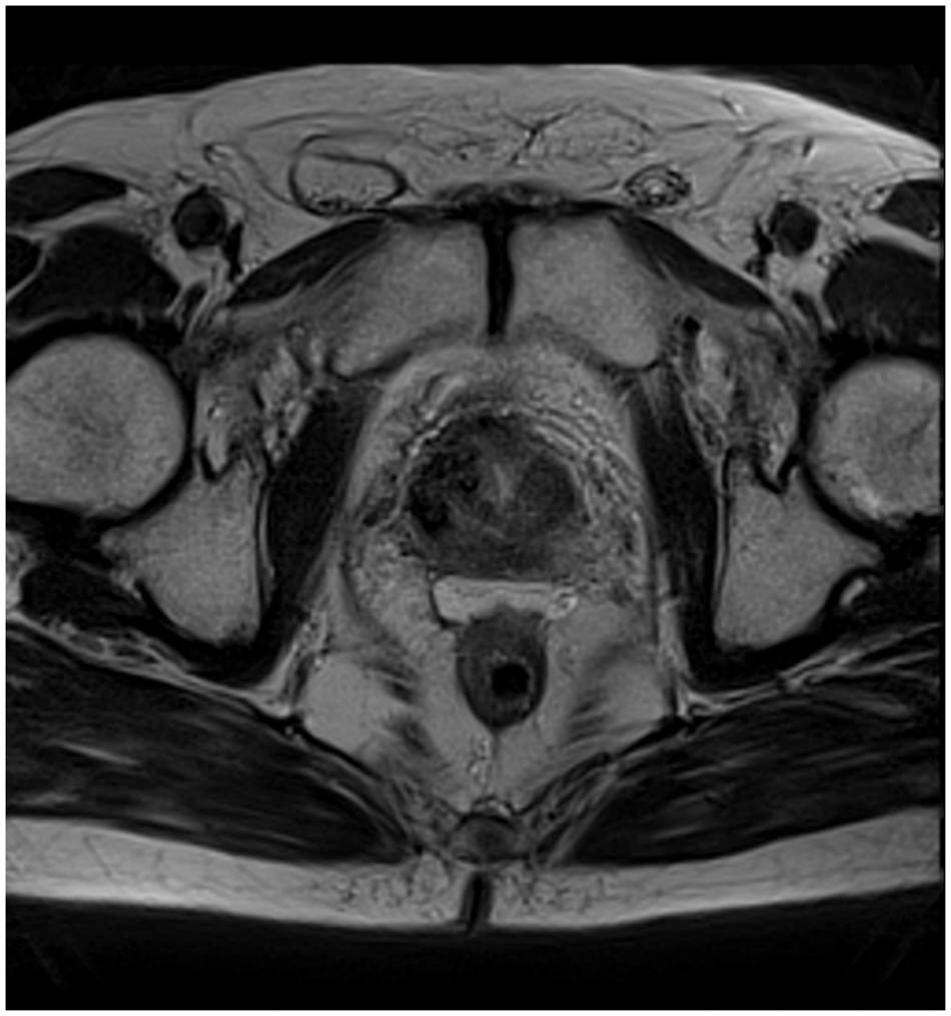
Selected axial MR image of the pelvis 3 months after salvage radiation therapy of recurrent prostate cancer showing anterior rectal wall thickening without fistula formation. The hydrogel SpaceOAR is in an optimal position between the prostate and the rectum, with symmetric distribution.

Following salvage brachytherapy, the patient experienced urinary symptoms, including urgency and nocturia occurring 4-5 times per night, along with occasional haematuria. Additionally, he reported rectal discomfort, prompting further evaluation. Sigmoidoscopy revealed mild proctitis of the anterior rectum without evidence of fistula formation. Subsequent MRI of the pelvis conducted 3 months post-radiation therapy showed thickening of the anterior rectum without evidence of fistula formation or intrarectal injection (Figure 3). The patient’s urinary and rectal symptoms were managed conservatively over the following 3 months. His symptoms resolved over this period without intervention. These complications following SpaceOAR injection and radiation therapy were classified as a Grade 3 adverse event. He required admission to hospital and hospital-based investigations. He did not require any further intervention, and his symptoms resolved.

### Patient 4

An 82-year-old man with prostate cancer recurrence 18 years after initial treatment with EBRT. Biopsy showed Gleason 8 recurrence, which involved the left seminal vesicle. SpaceOAR was delivered without difficulty. Initially after radiation therapy of 27 Gy over 2 fractions, the patient was well with no symptoms. Three months after the radiation therapy, he reported increased urinary frequency and urinary incontinence. He was found to have a urethral stricture and underwent dilatation of this stricture, relieving his symptoms. This was categorized as a Grade 3 adverse reaction requiring intervention.

### Patient 5

A 69-year-old man with prostate cancer recurrence 6 years after his initial treatment with low dose-rate (LDR) brachytherapy. Biopsy confirmed Gleason 9 disease with involvement of the right seminal vesicle. He underwent hydrogel SpaceOAR injection followed by salvage brachytherapy (27 Gy over 2 sessions) in 2021. The injection of the spacer was uncomplicated. After the radiation therapy, he reported mild urinary symptoms with urinary frequency and nocturia, which settled over 4 weeks. These complications following SpaceOAR injection and radiation therapy were classified as a Grade 1 adverse reaction with mild symptoms requiring clinical observations without intervention.

### Patient 6

An 80-year-old man with recurrent prostate cancer 10 years after initial treatment with EBRT. Disease was confined to the prostate and Gleason 9 on biopsy. He underwent hydrogel SpaceOAR injection. The patient reported mild pelvic and rectal discomfort following the injection of the hydrogel spacer. Five days after the procedure he was admitted with systemic symptoms of fever and malaise, initially assumed to be procedure-related and treated with IV antibiotics. However, on admission, he was diagnosed with COVID-19. Blood cultures were negative, and there was no leucocytosis. It was deemed clinically that his symptoms were related to acute COVID-19 rather than the SpacerOAR injection. His pelvic symptoms were classified as a Grade 1 adverse reaction with mild symptoms requiring clinical observations without intervention. He underwent salvage brachytherapy 8 weeks after insertion of the spacer (27 Gy over 2 sessions), which he tolerated well without complication.

### Patient 7

A 71-year-old man was diagnosed with prostate cancer recurrence 9 years after his initial treatment with EBRT. He underwent injection of SpaceOAR hydrogel before salvage prostate brachytherapy (27 Gy over two sessions). This was completed successfully. He reported no adverse symptoms following the SpaceOAR insertion or radiation therapy.

### Patient 8

This 77-year-old male was diagnosed with prostate cancer recurrence 12 years after initial treatment with EBRT. He was found to have organ-confined recurrence. He had successful insertion of the hydrogel spacer. He underwent salvage prostate brachytherapy of 27 Gy over 2 sessions. This was completed successfully. He reported no adverse symptoms following the SpaceOAR insertion or radiation therapy.

## Discussion

Our review highlights the potential for severe complications after SpaceOAR injection in previously irradiated patients. Our Radiation Department had previously treated 30 salvage cases without SpaceOAR with no serious complications. We had been in communication with other institutions who reported using rectal spacers, both hyaluronic acid and SpaceOAR without complication, and we felt that it could benefit our patients. Having already treated almost 150 radiation-naive patients following SpaceOAR delivery and having reviewed our experience with using SpaceOAR in this cohort, we were satisfied with the safety profile of the product and also had an excellent level of user experience (2 users performed all the SpaceOAR injections).

We have reviewed all of our salvage cases after SpaceOAR in an attempt to understand why the 2 patients who developed life-altering complications did so. The first patient seemed to have an uncomplicated gel placement with a reasonable distribution and no intrarectal injection. However, on review, we could see an adhesion between the prostate and the rectum, which, in retrospect, could be seen on the pre-treatment MRI as a narrow hypointense soft tissue strand between the prostate and rectum (Figure 1A). The adhesion may have limited the dispersal of the SpaceOAR, thus increasing the pressure from the gel on the surrounding tissues, contributing physically to the breakdown of the rectal mucosa after irradiation. Once infection occurs around any foreign material, as happened in this case, it is very hard to treat. The combination of a foreign material and pre-existing fibrosis in the tissue limits the delivery of antibiotics to the nidus of the infection.

The second severe complication occurred in a patient who had both prior radiation and prior surgery. The combination of these 2 factors resulted in a very dense fibrotic space between the rectum and prostate, with a rectal stricture. Hydro-dissection produced only a small space and only a small amount of gel could be inserted. We have previously noted that gel may track back along the needle path immediately after delivery, especially if the needle is removed quickly before the gel solidifies.^3^ Injection of gel against high pressure in the target space is more likely to force gel to track back along the needle track. In this case, the gel almost certainly tracked back along the needle track into the anterior fibres of the perianastomotic fibrotic stricture.

We had noted rectal wall injection in 6% of our early (radiation-naive) cases. None experienced significant complications and all underwent radiation in the usual manner without time delay or sequelae. Previous radiation exposure induces fibrosis and alters the vascularisation of affected tissues.^9^ In patients who have had prior radiotherapy, the relatively fragile tissues may be more easily compromised by physical trauma such as the passage of a needle or by the presence of a foreign material, especially a firm non-malleable product such as SpaceOAR. In this case, the small gel collection (1 mL) in the anterior rectal wall may have contributed to the breakdown of the rectal mucosa and abscess formation during or after radiation. The infection did not heal with antibiotics and became an indolent chronic infection in the pelvis after radiation, subsequently extending along tissue planes to involve the pubic bones. In retrospect, this patient with both prior radiation and surgery was not a good candidate for SpaceOAR. There was a rectal stricture that slightly restricted probe movement and hydrodissection was difficult. This should have indicated to us that the tissues were too fibrotic to proceed with SpaceOAR injection.

In the 2 cases above (1 and 2), both the volume and the cumulative radiation dose are outliers within the series. While this may be a confounder in determining the exact cause of rectal wall injury, some resistance to gel delivery was noted by the operator during both procedures. There was a rectoprostatic adhesion in the first patient (noted retrospectively) and a rectal stricture in the second. It is likely that the combination of a relatively high radiation dose and the presence of a firm, non-malleable foreign body adjacent to the irradiated volume in a confined space increased the risk of rectal wall injury. Prior to the cases with SpaceOAR, we had treated 30 patients who had prior radiation with salvage therapy without incurring any significant rectal wall injury or complication. Therefore, we feel that the main causative factor in these cases must be the gel. We surmise that the physical pressure of the gel combined with the insult of the added radiation caused erosion of the rectal wall, resulting in sinus and abscess formation, leading to fistula in one case and chronic perineal infection in the second.

In conclusion, our experience with using SpaceOAR in patients who have had previous radiation has resulted in a serious complication rate of 25% (2 of 8 patients), and we have stopped using the product in this patient group. There are particular constraints that apply to the delivery of this product that make cautious delivery difficult and tailored injection impossible. As SpaceOAR must be injected over 10 s before it solidifies, it is an “all or nothing” procedure once the injection starts. The procedure cannot be stopped and restarted because the material solidifies quickly in the delivery system and no further injection is possible after 10 s. The needle position cannot be adjusted once the injection starts. One tries to prepare for this with careful hydro-dissection before gel placement, both to open and to assess the space. If the space does not open easily, symmetrically and widely, the gel should not be injected. However, even with symmetric hydro-dissection, the material itself may all flow to one side as it takes the path of least resistance. Asymmetric distribution of the gel is frequently not recognized during injection, as imaging during injection is in the sagittal midline plane, and one cannot therefore see if the gel is flowing to one side only. It is only after the gel has been injected that the lateral distribution can be evaluated. Furthermore, once the injection starts, the sonographic visibility of the gel diminishes because microbubbles with artefacts form in the gel.

We question whether SpaceOAR, which forms a firm ball similar to a rubber ball in consistency, is the most suitable material to insert into previously irradiated tissues. We recommend SpaceOAR should be avoided if the diagnostic MRI shows any adhesions between the prostate and rectum and that it should not be given unless hydrodissection can be performed with minimal pressure and the injected saline expands the rectoprostatic space easily and symmetrically.

Six of 8 patients had no serious complications after SpaceOAR injection. Therefore, the gel can be used safely in some patients. Through SpaceOAR users group meetings, we have had personal communication with users who reported to us that they had treated several patients with SpaceOAR prior to salvage brachytherapy with no complications. However, we have also had communication with other institutions who have also experienced fistula formation. Therefore, we urge other users to proceed with extreme caution in this patient group. It could be argued that the complications we experienced were due to poor patient selection, with one patient having a potentially detectable adhesion between the rectum and prostate and the second having had a poorly dissectable rectoprostatic space and a rectal stricture. However, the complications we encountered were so severe that in the absence of published data indicating its safety in better-selected patients, we remain reluctant to use the product in previously irradiated patients.

Other rectal spacers, such as hyaluronic acid, may prove to be safer to use in salvage cases. This material can be injected slowly and the needle may be repositioned several times to achieve a symmetric distribution. The material is more liquid in consistency and theoretically should put less pressure on the rectum. We have had personal communication with users who tell us that they have treated many patients with hyaluronic acid prior salvage brachytherapy without complication. However, to our knowledge, these data have not been published, and there is no evidence in the literature yet regarding the safety of hyaluronic acid in salvage patients.

While hydrogel spacers, specifically SpaceOAR, represent a significant advancement in prostate cancer radiotherapy and their safety and efficacy in radiation-naive men is well documented, including by ourselves, SpaceOAR application in patients with prior pelvic radiation therapy is fraught with challenges. Our review has shown life-changing complications in 2 of 8 cases, and we have suspended its use in this patient group. Future research should focus on developing tailored strategies to safely and effectively deliver malleable and softer spacers in this vulnerable patient population to optimise therapeutic outcomes while minimising the risk of adverse events. We await the publication of the experience of other institutions, but we feel it is important to report our experience for the benefit of other users and, especially, patients.

## Learning point

- Severe complications can occur with hydrogel spacers in patients with prostate cancer with prior radiation therapy.
